# Supplementary material for: The impact of the COVID-19 pandemic on mental health and well-being of people living with a long-term physical health condition: a qualitative study
Source: BMC Public Health. 2021 Oct 7;21:1801. doi: 10.1186/s12889-021-11751-3 (PMC8496145; doi:10.1186/s12889-021-11751-3)
Supplement: Supplementary file 1 — Additional file 1. [file 12889_2021_11751_MOESM1_ESM.docx]

**Supplementary Material: Interview Topic Guide**

*[Each section contained multiple prompts removed here to reduce word limit/length]*

**Ask to describe ‘normal life’**

**UNDERSTANDING AND ADHERENCE TO GUIDELINES**

- **At the moment, are you self-isolating (how long for, reasons for this) a key worker, working but not a key worker, social distancing/ ‘staying at home’**
- **What do you understand by the ‘social distancing’ advice that is being given – what does it mean to you?**
- **Have you been able to stick to the social distancing advice that has been given to your group? Please tell us about why/ why not?**

**LONG TERM CONDITION/CANCER**

- **How has Covid-19 had an impact on the [long term condition/cancer]**

**SOCIAL LIFE**

- **How would you describe your social life before the Covid-19 pandemic?**
- **How would you describe your social life now that social distancing measures have been brought in because of Covid-19? Please tell us about this**

**MENTAL HEALTH**

- **How do you feel about the changes that have been brought about by Covid-19?**
- **Have they had any impact on your mental health or wellbeing? Please tell us about these**
- **Have you been doing/ planning anything to help with this?**
- **Why are you doing/ not doing these things?**

**PROSPECTION**

- **Has the pandemic meant that you have any worries for the future?**
- **How are these different from the worries you had before?**
- **Will this change the way you live your life in future?**
- **Has this changed any of your priorities for the future?**
